# First Case Report of *Salmonella enterica* Serovar Haifa in a Patient from Mysore, Karnataka, India

**DOI:** 10.3390/idr18040088

**Published:** 2026-08-17

**Authors:** Chinchana Shylaja Eshwarappa, Mahadevaiah Neelambike Sumana, Yogeesh D. Maheshwarappa, Morubagal Raghavendra Rao, Vidyavathi B. Chitharagi, Neetha S. Murthy, Supreeta R. Shettar, Veerabhadra Swamy G S, G K Megha, Shruthishree S C

**Affiliations:** Department of Microbiology, JSS Medical College and Hospital, JSS Academy of Higher Education and Research, Mysuru 570015, India; chinchanaes@gmail.com (C.S.E.); myogeesh@jssuni.edu.in (Y.D.M.); morubagalrao@jssuni.edu.in (M.R.R.); vidyavathi@jssuni.edu.in (V.B.C.); neethamurthy@jssuni.edu.in (N.S.M.); supreeta.r.shettar@gmail.com (S.R.S.); veerabhadraswamygs@gmail.com (V.S.G.S.); gkmegha1998@gmail.com (G.K.M.); shruthimc91@gmail.com (S.S.C.)

**Keywords:** *Salmonella* Haifa, non-typhoidal *Salmonella*, zoonosis, gastroenteritis, India, multidrug resistance

## Abstract

Background: Salmonellosis is a major global public health concern, commonly caused by serovars such as *Salmonella enterica* serovar Typhimurium and *Salmonella enterica* serovar Enteritidis. Rare serovars, however, may represent underrecognized links between environmental reservoirs and human infection. *Salmonella enterica* serovar Haifa is a sporadic serotype primarily associated with livestock and environmental sources and has previously been reported in Indian poultry but not in human clinical cases to date. Case Presentation: A 70-year-old male with a history of type 2 diabetes, presented with acute watery diarrhea and dehydration. One week prior to symptom onset, the patient reported direct contact with cattle and poultry in a rural setting. Laboratory investigations revealed leucopenia and elevated procalcitonin (3.73 ng/mL). Stool culture yielded non-lactose fermenting colonies on MacConkey agar and H_2_S-producing colonies on Hektoen enteric agar. The isolate was identified via VITEK 2 and confirmed as *Salmonella enterica* serovar Haifa by the National Institute for Research in Bacterial Infections (NIRBI). Antimicrobial susceptibility testing (AST) revealed that the isolate showed resistance to ampicillin, ceftriaxone, and ciprofloxacin. The patient was successfully treated with a three-day course of intravenous Azithromycin (1 g) and achieved rapid clinical recovery. Conclusions: To the best of our knowledge, this case represents the first reported human infection caused by *S. Haifa* in India. The finding highlights the potential zoonotic risk of rare non-typhoidal *Salmonella* serovars and emphasizes the importance of surveillance, routine serotyping, and antimicrobial resistance monitoring within a One Health framework.

## 1. Introduction

Salmonellosis, a disease of major public health concern, is caused by bacteria of the genus *Salmonella*, which are among the leading causes of bacterial gastroenteritis and systemic infections worldwide. The genus *Salmonella* comprises two species, *S. ongori* and *S. enterica*, the latter of which includes six subspecies and over 2600 serovars [1]. Globally, *Salmonella* infections are estimated to cause approximately 93.8 million cases of gastroenteritis annually [2], posing a substantial health and economic burden, particularly in developing countries.

The clinical manifestations of salmonellosis range from self-limiting gastroenteritis, commonly caused by non-typhoidal *Salmonella* (NTS), to severe systemic infections such as typhoid fever [3]. NTS serovars like *Salmonella enterica* serovar Typhimurium and *Salmonella enterica* serovar Enteritidis are the predominant causes of human disease globally [4]. The primary transmission route of NTS to humans is through the consumption of contaminated food products—particularly those of animal origin such as meat, eggs, and unpasteurized milk—or through direct contact with infected livestock and domestic animals [2,5].

While a few major serovars dominate the global epidemiological landscape, the identification and characterization of rare serovars are equally important for understanding local disease dynamics and emerging zoonotic risks. *Salmonella enterica* serovar Haifa is one such rare serotype. It is considered a globally sporadic serovar, predominantly associated with animal reservoirs, especially poultry and cattle, as well as environmental sources such as water and soil [6,7,8]. Human infections caused by *Salmonella enterica* serovar Haifa are exceedingly uncommon and have been documented only sporadically, mainly in Europe and the Middle East [9,10,11]. Its rarity in human clinical isolates underscores its primarily zoonotic or environmental nature and suggests limited adaptation to human hosts.

In the Indian subcontinent, where food- and waterborne infections remain a major cause of morbidity and mortality, comprehensive surveillance of *Salmonella* is crucial. However, most surveillance programs focus on the common serovars, potentially overlooking the emergence of unusual or region-specific strains [12,13]. A structured literature search was conducted using PubMed, Scopus, Web of Science, and Google Scholar databases for studies published up to January 2026, using the keywords “Salmonella Haifa”, “*Salmonella enterica* serovar Haifa”, “Human”, and “India”. No prior reports of human clinical infection due to *Salmonella enterica* serovar Haifa from India were identified. Therefore, to the best of our knowledge, this appears to be the first documented human case reported from India. Given this context, the present case holds both public health and academic importance. We describe a 70-year-old male presenting with acute gastroenteritis, from whom *Salmonella enterica* serovar Haifa was isolated. This case represents a rare human infection caused by a serovar typically associated with environmental and animal sources, providing valuable insights into its clinical significance, antimicrobial resistance profile, and epidemiological relevance.

## 2. Case Presentation

A 70-year-old male patient from Mandya, Karnataka, with a medical history of type 2 diabetes mellitus, bilateral osteoarthritis of knees (Grade 4), and prostatomegaly, was admitted to the Department of Geriatrics with a three-day history of multiple episodes of loose stools and vomiting. The patient reported approximately 6 to 8 episodes of watery diarrhea per day, accompanied by generalized weakness and abdominal discomfort. There was no report of fever, hematochezia, or mucus in stool.

During clinical history-taking, the patient mentioned attending a family function at a relative’s rural residence approximately one week before the onset of symptoms. The visit involved direct and indirect contact with domestic livestock, particularly cows and poultry (hens), reared on the property. The setting was a small, mixed livestock farm typical of rural Karnataka. The patient had no history of foreign travel and denied the consumption of raw or undercooked meat or unpasteurized dairy products. This background suggests a possible epidemiological link between the infection and environmental reservoirs, particularly livestock, which are recognized sources of *Salmonella enterica* serovar Haifa [5,6]. Notably, no other family members who attended the event with the patient reported similar gastrointestinal symptoms. On admission, the patient was afebrile with the following vital parameters: pulse rate 112 beats per minute, blood pressure 140/80 mmHg, respiratory rate 18 breaths per minute, and oxygen saturation 96% on room air. The patient was conscious, oriented, and mild to moderately dehydrated, with slightly reduced skin turgor and dry mucous membranes. Abdominal examination revealed a soft and non-tender abdomen with normal bowel sounds. There was no hepatosplenomegaly or palpable masses. Initial hematological investigations revealed normocytic normochromic anemia (Hb: 10.1 g/dL) accompanied by leucopenia (TLC: 2600 cells/cumm). The procalcitonin level was elevated (3.73 ng/mL), indicating a possible bacterial etiology. Renal and liver function tests were within normal limits. Stool microscopy did not show any inflammatory cells, red blood cells, or parasitic forms. Urine microscopy revealed 8–10 pus cells per high-power field, whereas urine culture showed no bacterial growth even after 48 h of incubation. The patient did not report any urinary symptoms such as dysuria, urinary frequency, urgency, or suprapubic discomfort. In the absence of clinical features of urinary tract infection and with a sterile culture result, this finding was interpreted as most consistent with asymptomatic sterile pyuria or possible sample contamination. Therefore, it was considered unlikely to represent a clinically significant urinary tract infection and was deemed unrelated to the present episode of Salmonella-associated gastroenteritis. Blood cultures were also obtained as part of the diagnostic evaluation but did not yield any bacterial growth [Table 1].

A freshly passed stool sample was collected in a sterile, wide-mouthed, leak-proof container and transported promptly to the Microbiology laboratory under appropriate conditions. As the sample was processed without significant delay, no transport medium was used. All procedures were carried out as per departmental standard operating procedures following CLSI guidelines. The specimen was inoculated onto MacConkey agar, Hektoen enteric agar, and Selenite F broth (HiMedia Laboratories Pvt. Ltd., Mumbai, Maharashtra, India), followed by subculture onto blood agar, MacConkey agar, and nutrient agar (HiMedia Laboratories Pvt. Ltd., Mumbai, Maharashtra, India) after 6–8 h of incubation. The colonies were subjected to standard biochemical tests. Automated identification was performed using the VITEK^®^ 2 Compact system (bioMérieux, Marcy-l’Étoile, France) with GN identification cards. Antimicrobial susceptibility testing (AST) was carried out using the VITEK^®^ 2 Compact system with N-405 cards and confirmed by the disk diffusion method using antibiotic discs (HiMedia Laboratories Pvt. Ltd., Mumbai, Maharashtra, India) according to CLSI 2025 guidelines. All plates were incubated aerobically at 37 °C for 18–24 h. Post-incubation, on MacConkey agar, the isolate produced non-lactose fermenting, smooth, circular, pale colonies. On Hektoen enteric agar, green to bluish-green colonies with black centers were observed, indicative of hydrogen sulfide (H_2_S) production. On blood agar, colonies appeared smooth, circular, grayish, and non-hemolytic.

The colonies were subjected to manual biochemical tests and automated identification using the VITEK 2 Compact System (bioMérieux, Marcy l’Etoile, France) with the GN Identification Card. The isolate was oxidase-negative, indole-negative, and urease-negative, citrate-positive, and demonstrated an alkaline slant/acidic butt with abundant H_2_S production on Triple Sugar Iron agar. Agglutination testing with group-specific antisera (O4 and Hi) showed positive agglutination for Hi and O4 antisera. The VITEK 2 system identified the isolate as belonging to the *Salmonella* group. Antimicrobial susceptibility testing (AST) was performed using the VITEK 2 Compact System with the N-405 card and confirmed by the disk diffusion method according to CLSI (2025) guidelines. The isolate was sensitive to amoxicillin/clavulanic acid, cotrimoxazole, and azithromycin, and resistant to ampicillin, ceftriaxone, and ciprofloxacin. The isolate was subsequently referred to the National Institute for Research in Bacterial Infections (NIRBI), a WHO Collaborating Center for diarrheal disease research, for further characterization. Serotyping was performed using the conventional slide agglutination method with specific O (somatic) and H (flagellar) antisera following the Kauffmann–White classification scheme recommended by the World Health Organization. Based on the antigenic profile obtained, the isolate was confirmed as *Salmonella enterica* serovar Haifa.

Once the diagnosis of gastroenteritis due to non-typhoidal *Salmonella* (NTS) was established, the patient was treated with intravenous azithromycin (1 g) for three days. Supportive management included intravenous fluids to correct dehydration and electrolyte imbalance, followed by gradual reintroduction of oral feeding with maintenance of adequate hydration. The patient showed significant clinical improvement within 72 h, with resolution of diarrhea and stabilization of vital signs, and was subsequently discharged in a stable condition. A follow-up stool culture was performed after clinical recovery and showed no bacterial growth. While examining the psychosocial and exposure history to identify the possible source of infection, the patient reported no stress or mental health issues, indicating a stable psychosocial environment. The patient had no known hereditary conditions or family history of gastrointestinal disease and reported maintaining good personal and environmental hygiene. However, he had a history of recent travel to a rural area to attend a family gathering, where he had direct and indirect contact with cattle and poultry, representing a potential zoonotic exposure. There was no history of contact with individuals returning from abroad or other distant regions. Although the patient reported good hygiene practices, exposure through contaminated food, water, or shared sanitation facilities during travel or at the gathering cannot be completely excluded. On further enquiry, no similar gastrointestinal illness was reported among family members or other individuals in the village during the same period, suggesting that this case was most likely sporadic rather than part of a localized outbreak.

## 3. Discussion

This case highlights the clinical and epidemiological significance of *Salmonella enterica* serovar Haifa, an uncommon non-typhoidal *Salmonella* (NTS) serovar known to cause sporadic cases of gastroenteritis and occasionally invasive infections in humans. *Salmonella enterica* serovar Haifa was first identified several decades ago as one of the rare serovars isolated from food and environmental sources, with its distribution reported across various continents, including Africa, Asia, and Europe [14]. Unlike the globally predominant *Salmonella* serovars such as *S. Typhimurium* and *S. Enteritidis*, which account for the majority of human salmonellosis worldwide [15], *S. Haifa* has been infrequently associated with human illness and is more commonly recovered from animal or environmental reservoirs.

In 2005, Japan reported a fatal foodborne infection caused by *Salmonella enterica* serovar Haifa in a human, marking one of the earliest well-documented severe clinical cases linked to this serovar [16]. Subsequent studies have demonstrated the isolation of *Salmonella enterica* serovar Haifa from various food and environmental sources, reinforcing its zoonotic potential. For instance, a surveillance study conducted in Kampala, Uganda, during 2012–2013, reported *S. Haifa* among 32 identified *Salmonella* serovars isolated from environmental, livestock, and wastewater samples, albeit with a very low prevalence (<2%) [17]. In South Africa, a large-scale survey of retail meat and poultry products conducted in 2023–2024 detected *Salmonella enterica* serovar Haifa among multiple serovars such as *S.* Kentucky, *S.* Typhimurium, and *S.* Enteritidis, though the frequency of Haifa isolation remained extremely low [18]. Similarly, *S. Haifa* was reported from meat and meat product samples in another South African study (2023), where 2.84% of 1758 samples were *Salmonella*-positive, with Haifa appearing as one of the rare serovars [19].

In severe gastrointestinal infections, elevated procalcitonin (PCT) often occurs without positive blood cultures due to bacterial translocation. This “leaky gut” allows bacterial endotoxins like lipopolysaccharide (LPS) to enter the circulation and trigger the systemic release of host cytokines, such as tumor necrosis factor-alpha (TNF-α) and interleukin-6 (IL-6) [20]. These mediators stimulate the *CALC-1* gene to produce PCT in various tissues, even if living bacteria are absent from the blood or were cleared by prior antibiotic use [21]. Additionally, GI pathogens like *Salmonella* often cause only transient bacteremia, meaning that they may not be captured in a culture despite a persistent systemic inflammatory response [22].

Interestingly, a study conducted in Sohag City, Egypt (2016) identified *S. Haifa* in both human and poultry sources, including one human isolate from seven stool samples and several isolates from chicken carcasses [23]. This observation indicates a potential association between poultry reservoirs and human infections. Despite these sporadic reports, *S. Haifa* remains a rare cause of human salmonellosis globally. The majority of isolates continue to be recovered from food and environmental samples rather than clinical sources [24].

Prior to this human case, *S. Haifa* was already documented in animal reservoirs in India, specifically in poultry. A study on *Salmonella* prevalence in poultry and the related environment found *S. Haifa* to be the most abundant serotype (58.33% (7/12) of isolates) among poultry samples in a particular investigation, with the authors noting it was isolated and reported for the first time in India in 2015 [25]. The presence of this serovar in major food animal reservoirs, along with its demonstrated enterotoxigenic potential in animal models, suggests a possible zoonotic risk to humans that may remain underrecognized [11]. In the present case, the patient reported exposure to cattle and poultry, which may represent a potential source of infection; however, a definitive epidemiological link could not be established. The present case, to the best of current knowledge, represents the first report of *Salmonella enterica* serovar Haifa isolation from a human clinical specimen in India, specifically from the Mysuru region. Its identification underscores the importance of routine serotyping in uncovering the circulation of rare *Salmonella* serovars that might otherwise go unnoticed. The patient’s history indicated exposure to cattle and poultry, which is consistent with previously documented reports identifying animal reservoirs and contaminated food products as potential sources of infection [13].

Although non-typhoidal *Salmonella* infections are typically self-limiting, infections caused by uncommon serovars such as *S. Haifa* warrant attention due to their potential for misdiagnosis and underreporting. The detection of this serovar in India emphasizes the dynamic nature of *Salmonella* epidemiology and the need for sustained pathogen surveillance [4]. Several uncommon Salmonella serovars have been documented in Karnataka and surrounding regions, suggesting a diverse non-typhoidal *Salmonella* ecology with potential zoonotic interfaces. *S. Weltevreden* was implicated in a 2009 outbreak in a nursing hostel in Mangalore (Karnataka), affecting 34 female students [23], and was also reported in a recent case of myocarditis in Mysuru (2025), where the isolated serovar was Weltevreden [1]. *S. Wien*, another rare NTS, was isolated in 2008 from 10 gastroenteritis cases in a tertiary care hospital in Mangalore [25]. More recently, *S. Hvittingfoss* was isolated from a human case of acute gastroenteritis in a rural area of Mysuru (Sumana et al., 2024), representing the first documented Indian human infection caused by this serovar [26]. A ceftriaxone-resistant *Salmonella enterica* serovar Newport was also isolated from a human case of acute gastroenteritis in Mysuru (Sumana et al., 2024) [27]. Furthermore, a recent genomic analysis reported *S. Oslo* isolated from clinical samples in southern India (Dutta et al., 2025), underscoring that even seldom-seen serovars are currently in circulation [28].

Non-typhoidal *Salmonella* (NTS) is a leading cause of foodborne illness globally, and increasing antimicrobial resistance (AMR) has become a major threat to effective treatment (Feasey et al., 2012, global) [29]. Resistance to first-line agents such as ampicillin, ciprofloxacin, and third-generation cephalosporins has been increasingly reported from both human and food sources (Hendriksen et al., 2011, global surveillance) [30].

In India, resistance among NTS isolates shows considerable regional variation. A multicentric study by Jacob JJ et al. (2020) reported resistance to ampicillin in 18.6%, ciprofloxacin in 11.4%, and ceftriaxone in 5.7% of clinical NTS isolates [31]. Similarly, Behl et al. (2017, India) observed ampicillin resistance in 21%, ciprofloxacin resistance in 14%, and ceftriaxone resistance in 6% of *Salmonella* isolates from tertiary-care hospitals, with multidrug resistance noted in approximately 23% of isolates [32]. A recent systematic review by Marchello CS et al. reported pooled resistance estimates of 9.8% for ciprofloxacin and 9.9% for ceftriaxone among non-invasive NTS isolates, indicating gradual expansion of resistance to critically important antimicrobials in this region [33].

Outside India, surveillance data demonstrate similar concerns. In the United States, Medalla et al. (2017, USA) reported that approximately 12% of NTS infections were caused by isolates resistant to at least one clinically important antimicrobial, including ampicillin, ceftriaxone, and ciprofloxacin, with resistant infections associated with increased hospitalization rates [34]. A study from China by Ke et al. (2020, China) revealed resistance to “critically important” antimicrobials like ceftriaxone (a third-generation cephalosporin) and ciprofloxacin (a fluoroquinolone) remains lower but is steadily rising, often reported in the 5–15% range [35].

The role of food animals in the dissemination of resistant NTS is well-established. Varma et al. (2005, USA) demonstrated that antimicrobial-resistant *Salmonella* isolated from retail meats were significantly associated with human infections, supporting food-chain transmission [36]. Molecular comparison studies by Wu C et al. (2018, China) further showed overlapping resistance profiles between human and animal isolates, highlighting the contribution of zoonotic reservoirs to the spread of multidrug-resistant strains [37].

Therefore, continuous monitoring of *Salmonella* serovar distribution across food animals, environmental reservoirs, and human infections is essential to understand transmission dynamics and emerging resistance patterns. Strengthened surveillance systems, coupled with molecular typing techniques and detailed clinical history-taking, can aid in identifying infection sources and interrupting transmission chains. Furthermore, the implementation of stringent hygiene practices, robust food safety measures, and targeted public health education is critical to reducing the burden of non-typhoidal *Salmonella* infections, particularly among vulnerable populations such as children, the elderly, and immunocompromised individuals [38]. A key limitation of this study is the absence of environmental and animal sampling, which precluded confirmation of the source of infection and limited the ability to establish a definitive zoonotic link.

## 4. Limitation of the Study

A key limitation of this study is that environmental or animal sampling was not performed in the area (Yedaganahalli Mandya) where the patient had potential exposure. Consequently, the exact source of infection could not be microbiologically confirmed. Although the patient’s history indicated close contact with cattle and poultry, which are known reservoirs of *Salmonella* serovar Haifa, the absence of parallel microbiological investigation of livestock, poultry, feed, and environmental samples (such as water or soil) limits the ability to establish a definitive epidemiological link between the human isolate and the suspected environmental source. In addition, screening of accompanying family members was not performed. Although such screening and retrospective epidemiological tracking could have provided further insights into possible colonization or transmission dynamics, the case was identified retrospectively after the patient had recovered and returned home, making such investigations not feasible. Furthermore, molecular characterization methods such as polymerase chain reaction (PCR) or whole-genome sequencing (WGS) were not performed in the present study. While the isolate was serotyped at a national reference center using standard serological methods, the absence of molecular characterization limits further insights into the genetic profile and epidemiological relatedness of the strain.

## 5. Conclusions

To the best of our knowledge, this case represents the first reported human infection caused by *Salmonella enterica* serovar Haifa in India, isolated from a geriatric patient with acute gastroenteritis and significant underlying comorbidities. The isolate demonstrated resistance to ampicillin, ciprofloxacin, and ceftriaxone; however, the patient achieved rapid clinical recovery following appropriate antimicrobial therapy. This case serves as a sentinel event, affirming the circulation of this rare serovar in the Indian zoonotic reservoir and stressing the urgent need for enhanced One Health surveillance to monitor the emergence and antimicrobial resistance patterns of non-typhoidal *Salmonella* serovars.

## Figures and Tables

**Table 1:** Clinical presentation and initial laboratory investigations at admission.

Parameter	Findings	Reference Range
General condition	Afebrile, conscious, and oriented	—
Pulse rate	112 beats/min	60–100 beats/min
Blood pressure	140/80 mmHg	90–120/60–80 mmHg
Respiratory rate	18 breaths/min	12–20 breaths/min
Oxygen saturation	96% on room air	≥95%
Hydration status	Mild–moderate dehydration with reduced skin turgor and dry mucous membranes	—
Abdominal examination	Soft, non-tender abdomen; normal bowel sounds	—
Hepatosplenomegaly/masses	Not detected	—
Erythrogram		
Hemoglobin (Hb)	10.1 g/dL	13–16.5 g/dL
RBC count	3.56 million/µL	4.5–5.5 million/µL
Hematocrit (PCV)	29.9%	40–48%
MCV	84.1 fL	83–101 fL
MCH	28.3 pg	27–32 pg
MCHC	33.8 g/dL	31.5–34.5 g/dL
RDW	15.2%	11.6–14%
Leucogram		
Total leukocyte count	2600 cells/µL	4000–11,000 cells/µL
Neutrophils	82.6%	40–75%
Lymphocytes	12.1%	20–40%
Monocytes	5.1%	2–10%
Eosinophils	0%	1–6%
Basophils	0.2%	0–1%
Absolute neutrophil count	2150/µL	2000–7000/µL
Absolute lymphocyte count	310/µL	1000–3000/µL
Platelet count	1.79 lakh/µL	1.5–4.5 lakh/µL
Other investigations		
Procalcitonin	3.73 ng/mL	<0.5 ng/mL
Renal function tests	Within normal limits	Urea: 16.6–48.5 mg/dL; Creatinine: 0.7–1.3 mg/dL
Liver function tests	Within normal limits	AST/ALT < 50 U/L; Bilirubin < 1.2 mg/dL
Stool microscopy	No inflammatory cells, RBCs, or parasites	—
Urine microscopy	8–10 pus cells/HPF	0–5 cells/HPF
Blood culture	No growth	

## Data Availability

All datasets generated or analyzed during this study are included in the manuscript.

## References

[1] Sumana M.N., Rao M.R., Chitharagi V.B., Satyasai B., Shettar S.R., Swamy V.G., Eshwarappa C.S., Maheshwarappa Y.D. (2025). Myocarditis associated with *Salmonella enterica* serovar Weltevreden gastroenteritis in medical practitioner; case report from South India. Front. Med..

[2] El-Baz A.H., El-Sherbini M., Abdelkhalek A., Al-Ashmawy M.A. (2017). Prevalence and molecular characterization of Salmonella serovars in milk and cheese in Mansoura city, Egypt. J. Adv. Vet. Anim. Res..

[3] Karodia A.B., Shaik T., Qekwana D.N. (2024). Occurrence of *Salmonella* spp. in animal patients and the hospital environment at a veterinary academic hospital in South Africa. Vet. World.

[4] European Centre for Disease Prevention and Control (ECDC) (2024). Salmonellosis—Annual Epidemiological Report for 2022.

[5] Milton A.A.P., Agarwal R.K., Priya G.B., Athira C.K., Saminathan M., Reddy A., Aravind M., Kumar A. (2018). Occurrence, antimicrobial susceptibility patterns and genotypic relatedness of *Salmonella* isolates from captive wildlife, their caretakers, feed and water in India. Epidemiol. Infect..

[6] Muthumbi E.M., Mwanzu A., Mbae C., Bigogo G., Karani A., Mwarumba S., Verani J.R., Kariuki S., Scott J.A. (2023). The epidemiology of fecal carriage of nontyphoidal Salmonella among healthy children and adults in three sites in Kenya. PLoS Neglected Trop. Dis..

[7] Mathole M., Carroll L., Khabo-Mmekoa C., Mabogoane N., Matle I. (2024). Annotated genome sequences of Salmonella Haifa, Salmonella Bangkok, and Salmonella Reading, isolated from chicken meat in South Africa. Microbiol. Resour. Announc..

[8] Matle I., Pfukenyi D.M., Maphori N., Moatshe N., Nkabinde T., Motaung A., Schmidt T., Seakamela E., Mwanza M., Ngoma L. (2025). Monitoring, surveillance, antimicrobial resistance and genetic diversity analysis of non-typhoidal Salmonella in South Africa from 1960–2023 from animal and animal products. PLoS ONE.

[9] Abdelmalek S., Kadry M., Elshafiee E.A., Hamed W., Moussa I.M., Al-Maary K.S., Mubarak A.S., Hemeg H.A., Elbehiry A. (2019). Occurrence of *Salmonella* infection and antimicrobial susceptibility for local *Salmonella* isolates from different sources in a cross-sectional study. Ital. J. Food Saf..

[10] de Lima Júnior V.P. (2024). Construction, Characterization and Evaluation of Novel Self-Destructing Attenuated Adjuvant Salmonella Strains Designed to be Delivered In Ovo.

[11] Karabasanavar N.S., Madhavaprasad C.B., Gopalakrishna S.A., Hiremath J., Patil G.S., Barbuddhe S.B. (2020). Prevalence of Salmonella serotypes S. Enteritidis and S. Typhimurium in poultry and poultry products. J. Food Saf..

[12] Hyeon J.Y., Chon J.W., Hwang I.G., Kwak H.S., Kim M.S., Kim S.K., Choi I.S., Song C.S., Park C., Seo K.H. (2011). Prevalence, antibiotic resistance, and molecular characterizatio of *Salmonella* serovars in retail meat products. J. Food Prot..

[13] World Health Organization (WHO) (2018). Foodborne Disease Salmonella (Non-Typhoidal).

[14] Mughini-Gras L., Pijnacker R., Duijster J., Heck M., Wit B., Veldman K., Franz E. (2020). Changing epidemiology of invasive non-typhoid *Salmonella* infection: A nationwide population-based registry study. Clin. Microbiol. Infect..

[15] Majowicz S.E., Musto J., Scallan E., Angulo F.J., Kirk M., O’Brien S.J., Jones T.F., Fazil A., Hoekstra R.M. (2010). International Collaboration on Enteric Disease “Burden of Illness” Studies. Clin. Infect. Dis..

[16] Kaibu H., Higashine H., Iida K., Ueki S., Ehara H. (2005). A fatal food intoxication case due to *Salmonella enterica* serovar Haifa. Jpn. J. Infect. Dis..

[17] Afema J.A., Byarugaba D.K., Shah D.H., Atukwase E., Nambi M., Sischo W.M. (2016). Potential sources and transmission of *Salmonella* and antimicrobial resistance in Kampala, Uganda. PLoS ONE.

[18] Ramatla T., Ngoma L., Adetunji M.C., Mwanza M. (2024). Genomic characterization of non-typhoidal *Salmonella* serovars isolated from retail poultry meat in South Africa. Microbiol. Resour. Announc..

[19] Madoroba E., Magwedere K., Chaora N.S., Matle I., Muchadeyi F., Mathole M.A., Pierneef R. (2021). Microbial Communities of Meat and Meat Products: An Exploratory Analysis of the Product Quality and Safety at Selected Enterprises in South Africa. Microorganisms.

[20] Becker K.L., Snider R., Nylen E.S. (2010). Procalcitonin in sepsis and systemic inflammation: A harmful biomarker and a therapeutic target. Br. J. Pharmacol..

[21] Doddikoppad P., Joshi D.N., Shenoy B. (2022). Procalcitonin: In diagnosis of paediatric infections. Karnataka Paediatr. J..

[22] Watanabe Y., Oikawa N., Hariu M., Fuke R., Seki M. (2016). Ability of procalcitonin to diagnose bacterial infection and bacteria types compared with blood culture findings. Int. J. Gen. Med..

[23] Antony B., Dias M., Pinto H., Shetty A.K., Boloor R. (2009). Food poisoning due to *Salmonella enterica* serotype Weltevreden in Mangalore. Indian J. Med. Microbiol..

[24] Zheng G., Chen L., Dong G., Ye K., Liu Z., Wang L. (2026). Epidemiology, antimicrobial resistance transmission, and One Health-based prevention of non-typhoidal Salmonella and Campylobacter infections: An updated literature review. Front Microbiol..

[25] Antony B., Scaria B., Dias M., Pinto H. (2009). *Salmonella wien* from gastroenteritis cases encountered in Mangalore, India: A report of 10 cases and review of literature. Indian J. Med. Sci..

[26] Sumana M.N., Rao M.R., Rajshekar D., Karthik K., Shah N.K., Swamy V., Eshwar C.S., Maheshwarappa Y.D. (2024). The first-ever encounter with *Salmonella enterica* serovar Hvittingfoss causing acute gastroenteritis in India: A case report. Infect. Dis. Rep..

[27] Sumana M.N., Maheshwarappa Y.D., Rao M.R., Deepashree R., Karthik M.V.S.K., Shah N.K. (2024). Ceftriaxone-resistant *Salmonella enterica* serovar Newport: A case report from South India. Front. Public Health.

[28] Dutta P., Halder G., Ghosh M., Antony B., Denny P., Ganai A., Dutta S. (2026). Draft genome sequence–based genomic characterization of *Salmonella enterica* subsp. enterica serovar Oslo isolated from clinical samples in South India and its comparison with global isolates. Jpn. J. Infect. Dis..

[29] Feasey N.A., Dougan G., Kingsley R.A., Heyderman R.S., Gordon M.A. (2012). Invasive non-typhoidal *Salmonella* disease: An emerging and neglected tropical disease in Africa. Lancet.

[30] Hendriksen R.S., Vieira A.R., Karlsmose S., Lo Fo Wong D.M., Jensen A.B., Wegener H.C., Aarestrup F.M. (2011). Global monitoring of *Salmonella* serovar distribution from the World Health Organization Global Foodborne Infections Network (GFN): Data for the period 2001–2007. Foodborne Pathog. Dis..

[31] Jacob J.J., Solaimalai D., Muthuirulandi Sethuvel D.P., Rachel T., Jeslin P., Anandan S., Veeraraghavan B. (2020). A nineteen-year report of serotype and antimicrobial susceptibility of enteric non-typhoidal Salmonella from humans in Southern India: Changing facades of taxonomy and resistance trend. Gut Pathog..

[32] Behl P., Gupta V., Sachdev A., Guglani V., Chander J. (2017). Patterns in antimicrobial susceptibility of Salmonellae isolated at a tertiary care hospital in northern India. Indian J. Med. Res..

[33] Marchello C.S., Carr S.D., Crump J.A. (2020). A Systematic Review on Antimicrobial Resistance among *Salmonella* Typhi Worldwide. Am. J. Trop. Med. Hyg..

[34] Medalla F., Gu W., Friedman C.R., Judd M., Folster J., Griffin P.M., Hoekstra R.M. (2021). Increased Incidence of Antimicrobial-Resistant Nontyphoidal Salmonella Infections, United States, 2004–2016. Emerg. Infect. Dis..

[35] Ke Y., Lu W., Liu W., Zhu P., Chen Q., Zhu Z. (2020). Non-typhoidal *Salmonella* infections among children in a tertiary hospital in Ningbo, Zhejiang, China, 2012–2019. PLoS Neglected Trop. Dis..

[36] Varma J.K., Molbak K., Barrett T.J., Beebe J.L., Jones T.F., Rabatsky-Ehr T., Smith K.E., Vugia D.J., Chang H.G., Angulo F.J. (2005). Antimicrobial-resistant nontyphoidal *Salmonella* is associated with excess bloodstream infections and hospitalizations. J. Infect. Dis..

[37] Wu C., Yan M., Liu L., Lai J., Chan E.W., Chen S. (2018). Comparative characterization of nontyphoidal *Salmonella* isolated from humans and food animals in China, 2003–2011. Heliyon.

[38] Scallan E., Hoekstra R.M., Angulo F.J., Tauxe R.V., Widdowson M.A., Roy S.L., Jones J.L., Griffin P.M. (2011). Foodborne illness acquired in the United States—Major pathogens. Emerg. Infect. Dis..

